# Palliative care in terminally ill advanced chronic liver disease patients

**DOI:** 10.1007/s00508-024-02436-z

**Published:** 2024-09-10

**Authors:** Katharina Pomej, Eva Katharina Masel, Gudrun Kreye

**Affiliations:** 1 Division of Gastroenterology and Hepatology, Department of Medicine III, Medical University of Vienna, Vienna, Austria; 2 Division of Palliative Medicine, Department of Medicine I, Medical University of Vienna, Vienna, Austria; 3 Division of Palliative Medicine, Clinical Department of Medicine 2, Krems University Hospital, Karl Landsteiner Private University for Health Sciences, Mitterweg 10, 3500 Krems an der Donau, Austria

**Keywords:** Palliative care, Quality of life, Liver disease, Advanced chronic liver disease, ACLD

## Abstract

While mortality rates from advanced chronic liver disease (ACLD) are rapidly increasing, patients with an advanced disease stage have a comparable or even higher symptom burden than those with other life-limiting diseases. Although evidence is limited there is increasing recognition of the need to improve care for patients with ACLD; however, there are many limiting factors to providing good palliative care for these patients, including unpredictable disease progression, the misconception of palliative care and end of life care as being equivalent, a lack of confidence in prescribing medication and a lack of time and resources. Health professionals working with these patients need to develop the skills to ensure effective palliative care, while referral to specialized palliative care centers should be reserved for patients with complex needs. Basic palliative care, along with active disease management, is best delivered by the treating hepatologists. This includes discussions about disease progression and advance care planning, alongside the active management of disease complications. Liver disease is closely associated with significant social, psychological, and financial burdens for patients and their caregivers. Strategies to engage the discussion in multidisciplinary teams early in disease progression help to ensure addressing these issues proactively. This review summarizes the evidence on palliative care for patients with ACLD, provides examples of current best practice and offers suggestions on how disease-modifying and palliative care can coexist, to ensure that patients do not miss opportunities for quality of life improving interventions.

## Background

In the last three decades optimization processes in prevention, early detection and treatment have resulted in significant improvements for chronically ill patients [1]. Hence, patients with advanced chronic disease are increasingly being offered palliative care support. The World Health Organization (WHO) defines palliative care as “an approach that improves the quality of life of patients and their families facing challenges associated with life-threatening illness, whether physical, psychological, social or spiritual, by preventing and relieving suffering through early identification and flawless evaluation and treatment of pain and other problems” [2]. In the past, palliative care was usually used late in the course of illness and was usually associated with death, dying, and hospice care. In the last decade, palliative care has emerged as one of the top 10 fastest growing subspecialties in medicine [3].

In contrast to other life-limiting chronic diseases, standardized mortality rates for advanced chronic liver disease (ACLD) have increased by approximately 400%, since 1970 in the UK [1]. In the USA, mortality rates increased by 65% from 1999 to 2016, largely due to an increase in the prevalence of alcohol-related liver disease (ArLD) [4]. While patients with compensated advanced chronic liver disease (cACLD) have a median survival of over 12 years, each year approximately 5–7% of patients progress to decompensated ACLD (dACLD), resulting in a drop of survival rates to a median survival of only 2 years [5]. Over 70% of patients with ACLD die in hospitals, which is approximately 25% higher than the average for all deaths [6]. Furthermore, studies from Europe, the UK and the USA show that many patients with ACLD do not receive liver transplantation [7–12]. Despite these low transplantation rates, only few patients are referred to palliative care, very often in late stages of the disease [12–16].

Patients with ACLD have significant palliative care needs due to their high symptom burden, poor quality of life, frequent hospitalizations, and high mortality risks [17]. Therefore, the timely implementation of palliative care interventions in this vulnerable patient population is indicated.

### Update 2023 of the Lancet article on addressing liver disease in the UK

The Lancet Standing Commission on Liver Disease recommended that clinicians caring for patients with liver disease should balance prolonging life through optimal disease-specific treatment and providing high-quality palliative care for those that cannot be saved [1, 18]. Despite these recommendations, a significant disparity remains in the provision of palliative care and symptom-focused treatment for patients with ACLD, when compared to patients with other serious diseases [17].

In the USA, only 30% of patients with ACLD are referred to specialized palliative or hospice care, which often happens in late stages, even in the last days of life [14], when hospital-based interventions have been exhausted. In the UK about 70% of liver disease-related deaths occur in hospital, compared with less than 40% of deaths from cancer and other chronic conditions such as lung diseases [18]. These numbers are even higher in patients with ArLD, where in-hospital deaths occur in 80% of cases. While for some patients, death at home may be the preferred option, as patients and caregivers are in familiar surroundings, unnecessary invasive procedures are avoided and patients retain control [17] other patients with ACLD prefer in-hospital death, especially if they are socially isolated [17].

There are multiple barriers to providing palliative care in ACLD, including fluctuating courses of disease and prognostic uncertainty, unrealistic hopes in the context of liver transplantation, perceived incompatibility of transplantation together with palliative care, reluctance of clinicians to initiate palliative care discussions, as these are often misinterpreted as cessation of active treatment options, lack of palliative care specialists, and inadequate education of healthcare professionals at undergraduate and postgraduate levels [19]. Patients with ACLD are often medically and socially alienated and may experience complex psychosocial issues such as addiction and stigma, which can lead to reduced health behavior and resource allocation [20]. Furthermore, a lack of evidence-based interventions is adding to suboptimal palliative care in ACLD [17]. This is also due to unique challenges in conducting palliative interventional trials, as has recently been described in a review by Verma et al. [21]. Thus, interest representatives, stakeholders and funders should continuously support research that addresses current inequities in palliative care, to avoid deterioration of outcomes for this vulnerable patient population. Although palliative care has received little attention in previous guidelines, national panels in the UK and the USA have recently stressed the need for palliative intervention trials in ACLD patients [22, 23]. Recently, the national REDUCe 2 study (ISRCTN26993825) comparing long-term palliative abdominal drainage with repeated in-hospital ascites drainage in patients with refractory ascites due to ACLD, has been implemented in the UK. This is important as ascites remains the most common complication of cirrhosis requiring hospitalization [23] and the care of patients with dACLD often includes optimization of symptoms most commonly related to ascites [17]. Another trial, currently ongoing in the USA, the PAL-LIVER trial (NCT03540771), is a comparative effectiveness study of hepatologist-led versus specialist-led consultative palliative care for outpatients with advanced liver disease [17]. These studies will provide important data on the potential of palliative care interventions to improve outcomes for patients with ACLD [17].

In 2021 Austria counted a total of 356 facilities, including mobile palliative teams and other medical institutions that provided palliative and hospice care [24]. Although the number of institutions that provide palliative care has increased over the last years, personal resources are lacking, which is why there is a continuous need for initiatives to increase the number of palliative care specialists. In order to overcome this, educational programs of various degrees of specialization are offered by 13 institutions Austria-wide and additionally there were 3455 voluntary hospice and palliative care workers in 2021 who supported the 1313 palliative care specialists [24]. In other countries, for example in the UK, increased attention has been given to educating medical students and palliative care specialists by the implementation of several initiatives, including the emphasis on palliative care during gastroenterology training and setting up the “Special Interest Forum for Undergraduate Medical Education” by the Association of Palliative Medicine Association [17]. Beyond this, the number of hepatologists and palliative care physicians interested in this field is increasing. In England, this led to the establishment of the “End of Life Specialist Interest Group of the British Association for Study of the Liver” in 2017 [17]. Improved collaboration between hepatology and palliative care could also help to optimize the quality of life of patients with ACLD in Austria.

### The role of timely integration of palliative care in patients with ACLD

For patients with ACLD, the burden of physical symptoms is high and comparable to that of patients with lung and colon cancers [25]. A recent systematic review found that the most commonly reported symptoms in end-stage liver disease (ESLD) patients were pain, dyspnea, muscle cramp, erectile dysfunction, sleep disturbances, depression and anxiety [26]; however, these problems are rarely addressed by hepatologists [27].

The role of palliative care for patients with ESLD remains poorly understood, also due to the fact that most studies in this field focus on the use of palliative measures in patients with hepatocellular carcinoma (HCC) [6]. In a recent study, palliative care for patients with ESLD or HCC led to a reduction in costs, decreased the likelihood of in-hospital death and improved advanced planning for these patients [28]. In the USA the number of palliative care consultations for patients with dACLD increased from only 0.97% in 2006 to 7.1% in 2012 [29]. The problem of low palliative referral numbers has recently been addressed in a retrospective cohort study [30]. The authors demonstrated that palliative care consultation during hospitalization occurred in only 30.5% of acute-on-chronic liver failure (ACLF) patients, while higher grade ACLF, prior palliative care consultation and HCC were associated with higher rates of specialty palliative care consultation [30].

Potential barriers to discussing palliative care and referrals to specialized palliative care (SPC) include factors related to the patient, the physician and the service. For patients, poor understanding of the disease progression and the role of palliative care can lead to unrealistic expectations and unwillingness to participate early in advanced care plan (ACP) discussions and SPC services [31]. Furthermore, the onset of hepatic encephalopathy (HE), a common decompensation event in ACLD, disables patients to actively participate in ACP discussions [32]. Two recent surveys of SPC physicians, hepatologists, and gastroenterologists identified several factors contributing to the issue, including no clear criteria for referral to SPC, discomfort with the role of SPC physicians in shared care, belief that palliative care is synonymous with end-of life care, inadequate time for complex discussions, and uncertainty regarding appropriate medications for symptom control [19, 33]. Nevertheless, the vast majority of gastroenterology and hepatology specialists (86%) acknowledge that patients with ESLD benefit from early palliative care involvement and that initial discussions about ACP should be initiated by themselves as they are the responsible physicians rather than by SPC physicians [19].

The perception that palliative care excludes disease-modifying treatment and transplantation has been identified as an important barrier to timely initiation of core palliative care measures [33, 34]. Although liver transplantation represents a potential cure only few patients each year receive a transplantation in the UK, while several thousands of other patients die from chronic liver diseases [26]. Approximately 15% of patients actively listed for liver transplantation, die or drop out from the waiting list each year [35]. The evidence suggests that actively listed patients benefit from early SPC involvement, mainly due to a reduction in symptom burden and improvement of patients’ moods [36]. Improving the quality of life of these patients, as well as of those who are not eligible for liver transplantation, is an important factor in providing high-quality care [37]. Furthermore, studies have demonstrated a significant survival benefit for patients who are introduced to palliative care specialists early in the treatment, as they are more receptive of having these types of conversations than at a later timepoint when their disease has progressed [38].

### Palliative care in ACLD

The unpredictable nature of ACLD and the challenge of accurate prognosis [39, 40] have led to the concept of parallel planning [18]. This strategy recognizes the frequent need to actively manage complications, such as endoscopic screening for varices, while simultaneously preparing patients and their families for a possible health deterioration which may proceed rapidly and unexpectedly [36, 41–44].

There are different screening tools that help to identify patients with ACLD who have a particularly poor prognosis and might benefit from palliative care [45, 46]. Apart from the widely used Child-Turcotte-Pugh score (CPS) and the model for end-stage liver disease (MELD) score, there are various frailty measurement tools (e.g. clinical frailty scale and the liver frailty index) that measure frailty by physical strength and subjective fatigue. This is important because increased frailty reduces tolerance to withstand decompensations and thus increases the risk of mortality of patients with ACLD [47]. Early discussions about the prognosis and disease progression, even before the onset of decompensating events, ensures that patients and their families are better prepared for disease progression [32].

A recent literature review found that patients and caregivers often wish for more information about the condition and respective treatment options [48]. The authors advocate incorporating conversations about advance planning into routine care of patients with cirrhosis, by talking about uncertain disease progress and examining patients’ changing priorities for care over time [48]. They have created useful resources, including visual aids and question prompts, to help clinicians initiate conversations at various paces with information material in multiple formats, thus acknowledging the low health literacy of some patients; however, not all patients or their caregivers want to have end of life conversations in the context of advance care planning. As the need for information changes over time and particularly as the disease progresses, these discussions may be revisited at later timepoints [31, 49]. In this context it is important to highlight that palliative care should not be seen as the sole responsibility of palliative care teams, also because patients prefer talking to their treating physician about disease progression rather than with someone they do not know [32].

Despite limited resources and increasing demands, hepatologists should develop skills to provide basic primary palliative care to their patients, so that palliative care specialists can focus on complex cases where their experience and skills are most needed [49].

### Symptom management

Improving the quality of life of patients and their families is the cornerstone of palliative care. A recent study found that improved management of HE, ascites, and malnutrition had the greatest impact on patients’ quality of life [50]. Avoiding hospitalization has been identified as an important goal by patients, and educating and empowering patients and their caregivers to optimize symptom control can help to support this [51]. For many patients, successful palliation includes effective symptom control and relief from unnecessary burdens, such as polypharmacy, which is why medications and interventions should be regularly reviewed [52].

### Hepatic encephalopathy

Hepatic encephalopathy (HE) is distressing for patients and caregivers, especially because they often do not know that it is a possible complication of ACLD, until they experience it for the first time [53]. Treatment of overt and covert HE has been shown to significantly improve health-related quality of life (HRQOL) [54, 55]. Relatives are often the first to notice subtle changes in personality and memory function indicative of covert HE and should therefore be empowered to prevent and treat this condition. Unfortunately, one study found that only 6% of patients and their caregivers knew they were being treated for HE and understood how the treatment regimen worked [56]. Encouraging patients and caregivers to adjust laxative and/or single doses may help to improve the quality of life and prevent unnecessary hospitalizations [53]. Recent efficacy studies of rifaximin in combination with lactulose in clinical practice have reduced both the frequency and duration of hospital admissions [57, 58], thus confirming the results of previously published clinical trials. Therefore, early use of rifaximin should be recommended in patients with advanced disease showing signs of HE [59].

### Ascites

Approximately 60% of patients with liver cirrhosis develop ascites at some point [60], which has a significantly negative impact on HRQOL [61, 62]. Conservative management with diuretic treatment is the cornerstone of ascites management and holds an important role, although its use is often limited due to concerns about negatively impacting renal function or inducing electrolyte imbalance [6]. To prevent this laboratory tests are often performed; however, as part of a shared decision-making process it may be appropriate to take a pragmatic view and limit the frequency of monitoring laboratory parameters in end of life patients [6]. Nevertheless, the vast majority of ACLD patients will require or require regular therapeutic paracentesis and emergency admissions for paracentesis are burdensome and expensive. In England the implementation of day clinic paracentesis services led to a reduction in healthcare costs, a reduction in the length of in-hospital stays, and resulted in a lower likelihood of dying in hospital compared to patients receiving unplanned emergency paracentesis [63]. The results of the mentioned study illustrate that parallel planning is beneficia and that the optimal management of chronic diseases is an important component of palliative care. Long-term abdominal drainage offers another option for patients with ascites who are refractory to treatment, particularly those who are too frail to make use of regular outpatient paracentesis services. Preliminary evidence even suggests that the safety profile of long-term abdominal drainage is comparable to that of single-time large volume paracentesis [64, 65]. In this context, a German study found that ACLD patients with refractory ascites who receive a tunnelled peritoneal catheter have lower mortality rates and similar incidences of spontaneous bacterial peritonitis as patients with regular large volume paracentesis [66]. Furthermore, peritoneal catheters were linked to higher rehospitalization rates and a higher incidence of acute kidney damage and hyponatremia. As pointed out in another study, implantation of peritoneal catheters needs further exploration because topics such as the need for antibiotic prophylaxis, ideal daily drainage volumes and the importance of albumin substitutions remain unsolved [67]. Patients with ascites that is refractory to treatment have a median survival of less than 6 months, and open conversations about the balance of burdens and benefits of all therapeutic options will help physicians to focus on individualized patient care priorities [68]. Importantly, palliative concepts, including implanted peritoneal catheters, do not have to exclude a potentially curative treatment such as liver transplantation. Furthermore, the alfapump®, a battery-powered pump designed to remove fluid from the peritoneal cavity into the bladder, offered the possibility for ascites drainage in an out-of-the hospital setting; however, this device is no longer available. Finally, data from a recently performed randomized controlled trial underline the need for further research in the field of ascites drainage devices and technologies, as they suggest that patients who had implanted fluid removal medical devices had improved HRQOL scores at 3 months, compared to patients who underwent multiple single-time large volume paracentesis [69].

### Malnutrition

It is increasingly recognized that frailty predicts poor outcomes in patients with advanced chronic liver disease, and sarcopenia is a common condition in these patients [70, 71]. As overnight fasting in these patients has similar effects to a 72‑h fast in healthy individuals [72], the European Association for the Study of the Liver (EASL) has published comprehensive guidelines on nutritional management [68] including simple measures, such as encouraging patients to snack before bedtime as they have been shown to improve the HRQOL [73]. There is also increasing evidence that optimizing diet in patients with covert HE can improve cognitive performance as well as HRQOL [50]. Therefore, early referral to a dietitian is important and all members of the healthcare team should educate patients and caregivers about the benefits of an optimized diet.

### Pain therapy

Despite a high pain and symptom burden, inadequate analgesic therapy is often reported by ACLD patients [74, 75]. One reason for this is the widely encountered concern about metabolism and side effects of medications, particularly analgesics, in advanced chronic liver disease, and recommendations for safe dosages are typically vague and encourage caution for prescribers [33]. If improving the QOL is the priority of care, it is of importance for patients, caregivers, and physicians to discuss the pros and cons of analgesics versus their potential side effects, such as worsening of HE. To improve consistency and safety in prescribing analgesics, the British Association for the Study of the Liver (BASL) end-of-life special interest group recently published pragmatic guidelines for pain and symptom control in ACLD (https://www.basl.org.uk/index.cfm/content/page/cid/33). In brief, the guidelines suggest avoiding nonsteroidal anti-inflammatory drugs, tramadolol, codeine and amitriptyline, while reduced doses of paracetamol and oral opioids like morphine sulphate and hydromorphone are safe first choice options in these patients. Although paracetamol seems to be the most feared drug by clinicians and ACLD patients, the evidence suggests that paracetamol intake in reduced doses (i.e., 2–3 g per day) is safe even in patients with alcoholic liver disease and can therefore be used as first-line medication in these patients [76]. Furthermore, nonpharmacological treatment such as acupuncture, massage and cognitive behavioral therapy can be considered [77].

### Coordination of patient care

General practitioners (GP) usually want to be closely involved in the care of patients with ESLD but providing community-based services can be difficult if the social circumstances of patients are challenging [78]. A recent British qualitative study of GPs pointed out concerns such as the lack of expertise in the field of hepatology, limited confidence in prognosis evaluation, and the desire for continued support from secondary/tertiary care facilities [41]. Similarly, patients expressed mixed experiences with the involvement of their GPs in the management of their liver disease, ranging from being supportive and interested, to lacking confidence and knowledge about ACLD in a systematic literature review [79]. To build a bridge between primary and secondary care, the implementation of a supportive liver care nurse has been studied in a recent feasibility study from Edinburgh [80]. The goal was to improve care coordination, ACP and QOL for patients with ESLD and their caregivers. The intervention was widely accepted, led to improvement of ACP and resulted in various potential financial benefits, such as the reduction of unplanned admissions, shorter hospital stays, and fewer primary care consultations [80]. Similar models to improve integration of primary and secondary care have been established, as illustrated in other UK case studies [6].

### Place of death

Increasing evidence suggests that while patients and caregivers may express a preference for death at home, the choice of location turns out to be far less important than comfort [81]. Indeed, death in hospital represents a preferred option for some patients with ESLD, particularly those with unstable social situations; however, there are discrepancies between the proportion of patients with CLD (78%) and patients with HCC (39%) who die in-hospital, which suggests that early discussions about the place of death occur less frequently in patients with benign ESLD [6]. Unfortunately, many patients with ESLD do not have a caregiver, which can become a source of concern for those patients as the disease progresses. Thus, it is important to explore alternative support options early as disease progresses and these discussions should be part of the core palliative care.

### Financial needs

In interviews both patients and caregivers often mentioned the financial burden, which is imposed upon them by the disease [51]. Most caregivers of patients with CLD are partners or spouses and a significant number reported that they have to reduce or even give up working hours due to the high caregiving demands [82, 83]. To offer some financial support, some hospitals have specialized teams to assist patients in applying for various social services. If these services are not available it is important for physicians to be aware of local services or nonprofit organizations that can help CLD patients with this complex process.

### Psychosocial treatment

Psychosocial treatment plays a crucial role in palliative care for terminally ill patients with ACLD. In addition to addressing the physical symptoms of the disease, psychosocial interventions focus on improving the patient’s overall quality of life and well-being. In the context of ACLD, these treatments include emotional support, coping strategies and interventions to manage depression, anxiety and existential distress. These interventions are important because there are high rates of depression (16%) and anxiety (43%) among these patients, as demonstrated in a multicenter observational study from 2020 [84, 85]. This holistic approach recognizes the complex interplay between physical symptoms and psychosocial factors such as social support, cultural beliefs, and existential concerns. By providing a supportive environment and addressing psychological and social needs by psychologists, social workers and volunteers, psychosocial treatment aims to optimize patient comfort, dignity, and emotional resilience.

### Do not forget the caregivers

Although positive aspects of caregiving are reported it is an extremely demanding role on individuals who in most cases have never received a formal training [86]. Factors that increase the burden of caregivers for patients with ACLD include a history of HE, multiple hospitalizations, active alcohol abuse, additional dependents in the household and low household income [83].

**Fig. 1:**
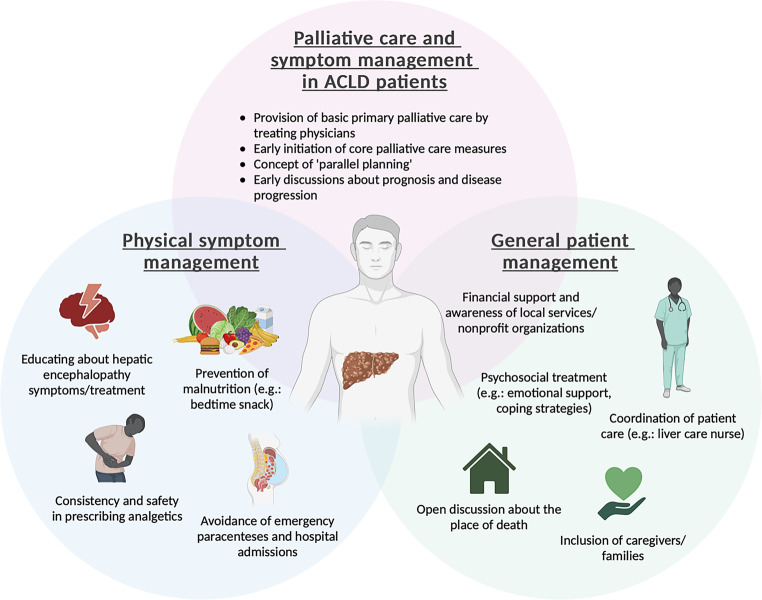
The role of palliative care in the management of terminally ill advanced chronic liver disease patients (ACLD). (Image created with biorender.com)

It is important for caregivers to know what local support services are available and what financial assistance they may be able to receive. This recognition helps to understand how important and challenging their role is in the care of patients with ACLD. Patients often want caregivers to be involved in advance planning discussions as for them it is reassuring to know that their caregiver knows their wishes and can be an effective advocate, especially when the time has come when they are no longer able to speak for themselves [32]. Studies have also shown that caregivers are better able to cope with grief when they know that their loved ones have received prolonged care from palliative care teams prior to death ([87]; Fig. 1).

## Future perspectives

Liver disease is a leading cause of death worldwide and tends to affect people at a younger age than other major causes of death (e.g. malignant diseases). Palliative and end-of life care for these patients is often neglected. Guidelines are emerging on what good palliative care for people with CLD should look like, but they often lack a patient perspective, even though they are the ones most affected by these guidelines. Limited palliative care resources mean that hepatologists must be responsible for basic palliative care as they are best placed to identify and support patients who might benefit from ACP discussions [6]. To achieve this, gastroenterologists and hepatologists in training, as well as specialist nurses, need to receive decent practical training in the principles of palliative care [6]. Thus, a proactive and methodical approach to concurrent planning, including early discussions about advance care planning, can help improve the situation for patients with ACLD [6].

## Abbreviations

ACLD: Advanced chronic liver disease
ACLF: Acute-on-chronic liver failure
ACP: Advanced care planning
ArLD: Alcohol-related liver disease
BASL: British Association for the Study of the Liver
cACLD: Compensated advanced chronic liver disease
CPS: Child-Turcotte-Pugh score
dACLD: Decompensated advanced chronic liver disease
EASL: European Association for the Study of the Liver
ESLD: End-stage liver disease
GP: General practitioner
HCC: Hepatocellular carcinoma
HE: Hepatic encephalopathy
HRQOL: Health-related quality of life
MELD: Model of end stage liver disease
QOL: Quality of life
SPC: Specialized palliative care WHO World Health Organization
